# The kinetics of SARS-CoV-2 viremia in COVID-19 patients receiving remdesivir

**DOI:** 10.1007/s10096-023-04627-4

**Published:** 2023-05-27

**Authors:** Anders Krifors, Linda Karlsson, Martin Ekman, Camilla Lorant, Paul Skorup

**Affiliations:** 1 Department of Physiology and Pharmacology, Karolinska Institutet, 171 65 Stockholm, Sweden; 2 Centre for Clinical Research Västmanland, Uppsala University, Hospital of Västmanland, 721 89 Västerås, Sweden; 3 Department of Infectious Diseases, Akademiska University Hospital, 753 09 Uppsala, Sweden; 4 Department of Clinical Microbiology, Karolinska University Hospital, 141 52 Stockholm, Sweden; 5 Department of Medical Sciences, Section of Infectious Diseases, Uppsala University, 751 85 Uppsala, Sweden

**Keywords:** COVID-19, Remdesivir, SARS-CoV-2, Viremia, Viral kinetics

## Abstract

Detection of SARS-CoV-2 RNA in serum, viremia, has been linked to disease severity and outcome. The kinetics of viremia in patients receiving remdesivir has not been thoroughly studied and could help predict treatment response and outcome. We investigated the kinetics of SARS-CoV-2 viremia and factors associated with baseline viremia, viral clearance and 30-day mortality in patients receiving remdesivir. An observational study including 378 hospitalised patients (median age 67 years, 67% male) sampled with serum SARS-CoV-2 RT-PCR within ± 24 h of initiation of remdesivir treatment. Baseline viremia was present in 206 (54%) patients with a median Ct value of 35.3 (IQR = 33.3–37.1). In patients with baseline viremia, the estimated probability of viral clearance was 72% by day 5. Ct values decreased significantly during remdesivir treatment for viremic patients, indicating an increase in viral load. In total, 44 patients (12%) died within 30 days, and mortality was significantly associated with viremia at baseline (OR = 2.45, p = 0.01) and lack of viral clearance by day 5 (OR = 4.8, *p =*  < 0.01). Viral clearance was not associated with any individual risk factor. Viremia appears to be a prognostic marker before and during remedesivir treatment. The resolution of viremia was similar to patients not receiving remdesivir in other studies, and the decrease in Ct values during treatment questions the antiviral capacity of remdesivir in vivo. Prospective studies are warranted to confirm our findings.

## Introduction

Detection of SARS-CoV-2 RNA by real-time PCR (RT-PCR) in serum, viremia, has been reported to correlate with disease severity, ICU admission and mortality, and may be used as a predictor for the clinical outcome [1-5]. Therefore, viremia could be a marker of uncontrolled viral replication and play a significant role in the pathogenesis of severe COVID-19. Further, disseminated viral particles from the lungs could drive the dysregulated inflammatory host response seen in critical COVID-19 patients [6].

Remdesivir is a nucleoside analogue (GS-5734) that inhibits RNA polymerase activity and was initially developed for the Ebola virus. Remdesivir was the first antiviral drug approved for the treatment of COVID-19 [7] and has demonstrated potent antiviral activity against the SARS-CoV-2 virus in vitro, including emerging variants of concern [8-10]. Despite promising data from in vitro studies and early primate models [11], subsequent clinical trials have reported conflicting results. For example, the ACTT-1 and Spinner trials [12, 13] did not demonstrate any survival benefit or impact of remdesivir on viral shedding. However, in both trials, the median symptom duration at the treatment start exceeded seven days. At this time, viral replication is expected to be low, limiting the use of an antiviral agent. The WHO-sponsored SOLIDARITY trial [14], the most extensive study to date, demonstrated a slight benefit in mitigating disease progression and improving survival in patients without mechanical ventilation. However, the duration of symptoms at the treatment start was not reported.

Growing evidence shows that remdesivir does not contribute to viral clearance in the upper respiratory tract [15-17]. Nevertheless, viremia may be a better marker of clinically relevant viral replication due to the reported association with disease progression and mortality [1, 18]. There is a lack of studies on the kinetics of SARS-CoV-2 viremia in patients receiving remdesivir. This study aims to bridge this gap by investigating retrospectively the prevalence and kinetics of SARS-CoV-2 RNA in serum among COVID-19 patients receiving remdesivir. A secondary aim of this study was to identify factors associated with baseline viremia, lack of viral clearance and mortality at 30 days.

## Materials and methods

This retrospective cohort study included all hospitalised COVID-19 patients ≥ 18 years receiving remdesivir between 1 July 2020 and 30 April 2021 at Akademiska University Hospital, a tertiary care hospital in Uppsala and Västerås Hospital, a regional hospital in Västerås, Sweden. The inclusion criteria were the measurement of serum SARS-CoV-2 by RT-PCR within + / − 24 h of initiation of remdesivir treatment. At the study centres, serum SARS-CoV-2 RT-PCR testing was recommended at the initiation of remdesivir to evaluate the virological response and was typically performed every 2–3 days at the attending physician’s discretion. Serum from peripheral blood was collected from the patients, and viral kinetics was determined by sequential serum SARS-CoV-2 RT-PCR testing.

At the Department of Microbiology at the Karolinska University Hospital, Huddinge, Stockholm, the following commercial techniques approved for diagnosing SARS-CoV-2 were used: cobas SARS-CoV-2 assay (Roche, Pleasanton, California), targeting the Envelope (E) and Open Reading Frame (ORF) 1 gene [19] or NeuMoDx SARS-CoV-2 assay (NeuMoDx, Ann Arbor, Michigan) targeting non-structural protein 2 (Nsp 2*),* RNA-dependent RNA polymerase* (*RdRp*) *and nucleocapsid (N) genes. In addition, a validated in-house method targeting the RdRp and E-genes was utilised. At the Department of Microbiology at Akademiska University Hospital, Uppsala, a validated in-house method targeting the N1 and E genes was used. All analyses were performed per the manufacturer’s instruction and under standard clinical practice. Cycle threshold (Ct) values were used as a surrogate marker of SARS-CoV-2 viral load. In cases of multiple Ct values, the target gene with the lowest Ct value was chosen and used for subsequent samples. Ct-values > 40 were considered negative and were not included in analyses involving Ct values. Ct values over time were analysed with linear regression.

The primary analyses were viremia at the start of remdesivir treatment, viral clearance, and mortality at 30 days. Clinical and physiological data and baseline characteristics were acquired from the patient’s medical records and reported as medians, interquartile range, frequency, and percentage for continuous and categorical variables, respectively. The risk factors associated with severe COVID-19 were adopted from the Center for Disease Control and Prevention [20].

### Statistical analysis

STATA version 16.1 (StataCorp, Texas, USA) software was used for the statistical analyses. Baseline characteristics were assessed using the Shapiro-Wilk test for normality, and results were presented as median and interquartile range (IQR) when normality could be rejected at a 0.05 significance level. Logistic regression was used to analyse risk factors associated with baseline viremia and 30-day mortality and presented as odds ratios with an alpha significance level of 0.01 to account for multiple testing. Viral clearance was estimated using the Kaplan-Meier survival analysis. Cox regression was utilised to investigate the impact of a set of clinically relevant predictor variables on the time to achieve negative viremia, presented as hazard ratios with an alpha significance level of 0.01. Ct-values over time were analysed using linear regression.

The study was performed following the guidelines of the Declaration of Helsinki and approved by the Swedish Ethical Review Board, Stockholm (Dnr 2021-01878). The Swedish Ethical Review Board waived the need for informed consent.

## Results

Remdesivir treatment was initiated in 435 patients during the study period, of whom 378 had a measurement of serum SARS-CoV-2 within ± 24 h of remdesivir treatment start and were included in the study. The baseline characteristics of the study population, including 254 (67%) men and 124 (33%) women, are presented in Table 1. The median age was 67 years, and the median BMI was 28 kg/m^2^. The most common risk factors were hypertension (*n* = 240, 63%), BMI > 30 kg/m^2^ (*n* = 166, 44%) and diabetes mellitus (*n* = 116, 31%). The patients had a median symptom duration of seven days (IQR = 5–8 days) at the start of remdesivir therapy, and 338 (89%) patients required supplemental oxygen at the start of remdesivir treatment.

**Table 1:** Baseline Characteristics including univariate analysis for baseline viremia

Study Population	All patients (*n* = 378)	Patients with baseline viremia (*n* = 206)	*p*-Value*
Age, median (IQR), years	67 (55–78)		0.40
Age > 65 years (%)	206 (54)	111 (54)	0.60
Body Mass Index, median (IQR), kg/m^2^	28 (25–32)		0.13
Body Mass Index > 30 kg/m^2^ (%)	166 (44)	80 (48)	0.03
Sex, male, n (%)	254 (67)	143 (56)	0.31
Hypertension, n (%)	240 (63)	130 (54)	0.87
Immunosuppression, n (%)	74 (20)	39 (53)	0.75
Chronic Kidney Disease, n (%)	22 (6)	10 (45)	0.38
Chronic Liver disease, n (%)	9 (2)	5 (56)	0.95
Diabetes mellitus, n (%)	116 (31)	70 (34)	0.13
COPD, n (%)	68 (18)	36 (60)	0.78
Cardiovascular disease, n (%)	62 (16)	37 (60)	0.37
Heart failure, n (%)	47 (12)	25 (53)	0.85
At least one risk factor, n (%)	337 (89)	188 (56)	0.15
Supplemental oxygen, n (%)	338 (89)	198 (59)	< 0.01
Nasal cannula	281 (83)		
NIV or HFNC, n (%)	53 (16)		
Invasive Mechanical Ventilation, n (%)	4 (1)		
Respiratory rate, median (IQR)	24 (20–28)		0.05
Oxygen saturation, median (IQR), %	94 (91–95)		0.01
Symptom duration, median (IQR), days	7 (5–8)		0.89
C-reactive protein, median (IQR) mg/L	89 (46–149)		< 0.01
C-reactive protein, > 100 mg/L	198 (52)	123 (62)	< 0.01
Detectable serum SARS-CoV-2 RNA, n (%)	206 (54)		
Ct value, median (IQR)	35.3 (33.3–37.1)		

*IQR*, interquartile range, *NIV*, non-invasive ventilation, *HFNC*, high flow nasal cannula, *COPD*, chronic obstructive pulmonary disease

* Univariate analysis using logistic regression

Baseline viremia was detected in 206 (54%) patients with a median Ct value of 35.3 (IQR = 33.3–37.1). There was a strong association between baseline viremia and CRP > 100 mg/L (OR 1.9, *p =*  < 0.01) and supplemental oxygen (OR 5.66, *p =*  < 0.01), but no association was found with symptom duration. The median duration of remdesivir treatment was five days (IQR = 4–5 days).

**Fig. 1:**
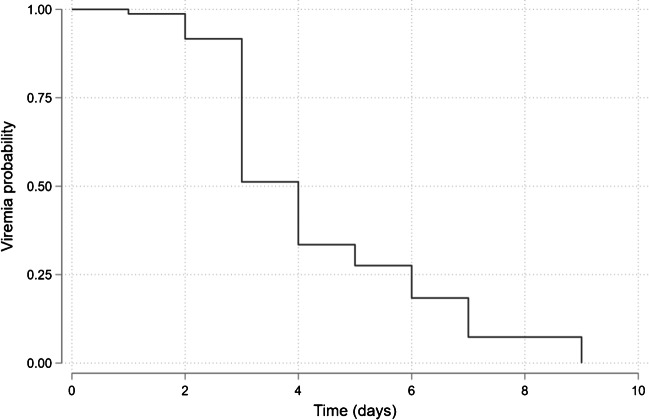
SARS-CoV-2 viremia probability, Kaplan-Meier curve

**Fig. 2:**
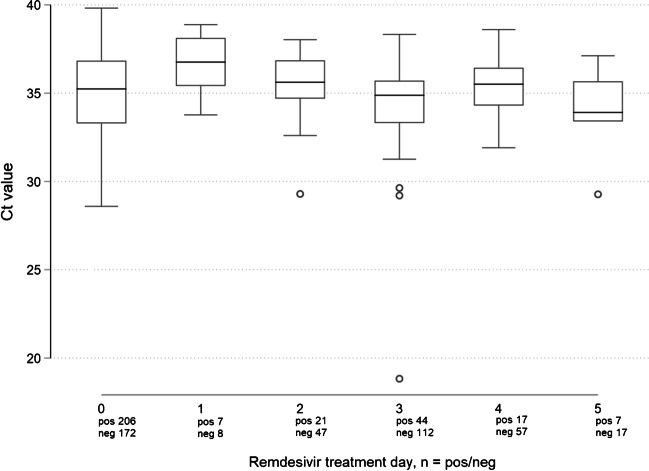
Ct values during remdesivir treatment including number of positive/negative tests. Boxplot median, interquartile range, max-min and outliers

A second SARS-CoV-2 RT-PCR was performed in 271 (72%) patients, and out of them, 76 (20%) patients were sampled thrice. In patients with baseline viremia, the Kaplan-Meier probability of viral clearance was 72% by day 5 (Fig. 1). The median duration of symptoms at viral clearance was ten days (IQR = 8–11 days). There were no significant associations between viral clearance and individual risk factors (Table 2). Ct values decreased significantly throughout remdesivir treatment (R^2^ = 0.03, *p =* 0.01) (Fig. 2).

**Table 2:** Association between risk factors and viral clearance, Cox regression

	HR (95% CI)	*p*-Value
Age > 65 years	0.99 (0.67–1.46)	0.97
Sex, male	0.66 (0.44–1.01)	0.05
Body Mass Index > 30 kg/m^2^	0.98 (0.66–1.46)	0.93
Hypertension	0.79 (0.52–1.18)	0.13
Immunosuppression	1.34 (0.84–2.13)	0.22
Chronic Kidney Disease	0.42 (0.10–1.70)	0.22
Chronic Liver disease	1.78 (0.44 –7.28)	0.42
Diabetes mellitus	0.70 (0.43–1.08)	0.11
COPD	0.85 (0.51–1.44)	0.55
Cardiovascular disease	0.85 (0.50–1.44)	0.55
Heart failure	1.03 (0.56–1.89)	0.92
At least one risk factor	0.84 (0.46–1.55)	0.59
C-reactive protein, > 100 mg/L	0.78 (0.53–1.16)	0.22

*HR*, hazard ratio, *CI*, confidence interval, *COPD*, chronic obstructive pulmonary disease

We also report the death of 44 patients (12%) within 30 days. Mortality at 30 days was significantly associated with age > 65 years (OR = 49.1, *p =*  < 0.01), viremia at baseline (OR = 2.45, *p =* 0.01) and cardiovascular disease (OR = 3.16, *p =*  < 0.01) (Table 3). Viremia at baseline remained a significant risk factor in a multiple logistic regression model, including age, sex and at least one risk factor (OR = 2.8, *p* < 0.01).

**Table 3:** Association between risk factors and 30-day mortality, logistic regression

		OR (95% CI)	*p*-value
30-day mortality, n (%)	44 (12)		
Age > 65 years		49.1 (6.68–360)	< 0.01
Sex, male		0.75 (0.39–1.43)	0.38
Body Mass Index > 30 kg/m^2^		0.62 (0.32–1.21)	0.17
Hypertension		2.11 (1.01–4.42)	0.05
Immunosuppression		1.94 (0.95–3.93)	0.07
Chronic Kidney Disease		2.39 (0.84–6.84)	0.10
Chronic Liver disease		0.95 (0.12–7.76)	0.96
Diabetes mellitus		1.67 (0.87– 3.18)	0.12
COPD		1.86 (0.90–3.84)	0.09
Cardiovascular disease		3.16 (1.58–6.33)	< 0.01
Heart failure		2.36 (1.08–5.17)	0.03
At least one risk factor		5.85 (0.78–43.7)	0.09
Baseline viremia		2.45 (1.22–4.92)	0.01
C-reactive protein, > 100 mg/L		1.51 (0.80–2.88)	0.21
Baseline oxygen therapy		5.68 (0.76–42.45)	0.09
Length of hospital stay, median (IQR), days	10 (7–16)		
Dexamethasone treatment, n (%)	207 (55)		
Antibiotic treatment, n (%)	174 (55)		

*IQR*, inter quartile range, *OR*, odds ratio, *CI*, confidence interval

In total, 207 (55%) patients received dexamethasone, and 174 (46%) received antibiotic therapy. The median length of hospital stay was ten days.

## Discussion

This observational retrospective study is the first to investigate SARS-CoV-2 viremia in patients treated with remdesivir. Remdesivir is still widely used partly due to the extensive drug interactions associated with the newer antiviral nirmatrelvir/ritonavir.

We found viremia to be an independent prognostic marker in remdesivir-treated patients due to the strong association with mortality. The association was unaffected when included in a multiple regression model including age, sex and at least one risk factor.

The probability of viral clearance by the end of remdesivir treatment (day 5) was only 72%, and the lack of viral clearance before day five was significantly associated with increased mortality. The median symptom duration at viral clearance was ten days, similar to a cohort study by Hagman et al. [1], where 24% (99/417) of hospitalised patients were viremic when sampled within three of admission and with a medium symptom duration of ten days. In another study by Ram-Mohan et al. [3], 52% (14/27) of COVID-patients had undetectable plasma SARS-CoV-2 RNA within ten days from the onset of symptoms, indicating resolution of viremia in a majority of patients within ten days regardless of remdesivir treatment.

In addition, we observed a significant reduction in Ct values indicating increased viral load among viremic patients throughout the treatment, which questions the antiviral capacity of remdesivir in-vivo*.* However, only patients that remained viremic were included in the analysis, which amplifies the effect. Two other studies support the lack of antiviral effect in vivo. Firstly, in the DisCoVeRy study [21], Ader et al. did not find any difference in the viral load in nasal swabs between the remdesivir group and the standard of care group, though a late administration of the drug (median nine days after onset of symptoms) partly weakens the result. Secondly, a recent study by Sourander et al. [15], investigated the kinetics of SARS-CoV-2 RNA in nasopharynx/throat swabs and found no significant difference in viral clearance between remdesivir-treated patients and controls.

Real-life data are vital to understanding how remdesivir is implemented under normal circumstances, which may differ substantially from clinical trials. We observed a median duration of symptoms of seven days at the start of treatment, at the upper limit of the Swedish national treatment guidelines and the WHO recommendations stating, “Remdesivir should be administered as soon as possible after the onset of symptoms, ideally within seven days [22, 23]. The treatment duration for the absolute majority of patients was five days, as recommended by the guidelines.

Baseline viremia was present in only 54% of the patients, and the high median Ct value of 35.3 is consistent with reports of the highest viral titres early in the first week of illness. We found no significant associations between baseline viremia and symptom duration. This discrepancy may partly be explained by a close clustering of most patients around a symptom duration of seven days, limiting the statistical power to detect a time-dependent association.

Previous studies have reported a positive association between viremia and disease severity [18, 24, 25]. In a prospective case series study involving 57 hospitalised COVID-19 patients, persistent viremia was found to be correlated with adverse clinical outcomes, potentially serving as an identifier for patients at risk [26]. Thus, an uncontrolled viral replication may induce and/or exacerbate a dysregulated host response, including cytokine storm, in patients with severe COVID-19. This hypothesis is supported by a study conducted by Rovito et al., which demonstrated increased pro-inflammatory cytokines, diminished activated T-cells, and reduced functional SARS-CoV-2-specific immune responses in viremic patients [27]. Another hypothesis is that inflammation-induced capillary leakage in the lungs may lead to the translocation of viral particles into the systemic circulation, which is detected by the RT-PCR analysis [28]. Viremia may reflect the disease severity but may not exacerbate the disease phenotype in these patients. We found an association between baseline viremia and 30-day mortality and a robust association between CPR levels above 100 mg/L and baseline viremia, consistent with both of these hypotheses.

Overall, a large set of variables were used to analyse the outcomes of 30-day mortality and viral clearance. To account for multiple testing and the risk of false positives, the significance level was lowered to an alpha significance level of 0.01.

The strength of our study is the inclusion of a relatively large number of patients receiving remdesivir in a real-life setting. Sequential viremia testing was available in most patients, 72% were tested twice, but only 20% were tested thrice. The co-administration of other COVID-19 medications, for example, dexamethasone, was present in 55% of the cases, further strengthening the clinical translatability of the results. However, due to the observational design of the study, it was not possible to evaluate the potential effects of steroids on viremia.

Limitations of our study include the retrospective design and lack of a control group restricting conclusions regarding the treatment effects. Also, testing was not conducted at standardised time points, limiting the number of patients where sequential testing was available. Viremia testing was conducted based on clinical judgement and, therefore, subject to confounding. For example, viral clearance could be underestimated due to missing follow-up viremia testing, potentially biased towards not testing clinically improving patients. The study design is not mechanistic, so any causal inference of the predictive capability of viremia should be cautiously interpreted. The majority of patients in our study were included from a period when SARS-CoV-2 vaccination was unavailable, and until the end of the study, vaccination rates remained low. As a result, the impact of immunisation on our findings is highly unlikely. However, the absence of vaccination status information for our cohort represents a limitation of our study.

Various PCR methods were used throughout the study and may not represent a single standardised method. Further, Ct values can differ significantly between assays and target genes, and drawing a solid conclusion regarding the evolution of Ct values in our data is difficult. We used Ct values as a surrogate marker for viral load but may not accurately represent the true viral load as some studies did not find a strong association between the Ct value and clinical markers or infectivity [29, 30]. Finally, patients included in the study were predominantly infected with the original strain and the alfa and delta variants of concern, and the results could potentially not be valid for current omicron variants.

In conclusion, SARS-CoV-2 serum RT-PCR appears to be a prognostic disease marker before and during remdesivir treatment. The resolution of viremia in remdesivir-treated patients was similar to untreated patients in other studies questioning the antiviral efficacy of remdesivir in treating COVID-19 infection. Clinical implications of the study include questioning the use of remdesivir in younger patients without baseline viremia, where the overall mortality appears to be low. Clinical prospective studies are warranted to investigate the effect of remdesivir on the kinetics of SARS-CoV-2 RT-PCR in serum.

## Data Availability

The data presented in this study are available from the corresponding author upon request, and the data are not publicly available due to ethical restrictions.
